# Psychosocial factors associated with medication adherence among older adults with chronic diseases: an ITHBC-informed cross-sectional study

**DOI:** 10.1038/s41598-026-58469-z

**Published:** 2026-06-16

**Authors:** Jingyue Guo, Peipei Pu, Teng Liu, Mingfen Wu

**Affiliations:** 1 Medical Affairs Department, Dahongmen Community Health Service Center, Fengtai District, Beijing, 100075 China; 2 Department of Pharmacy, Beijing Tiantan Hospital, Capital Medical University, No.119 South Fourth Ring West Road, Fengtai District, Beijing, 100070 China

**Keywords:** Medication adherence, Older adults, Chronic conditions, Psychosocial associated factors, Integrated theory of health behavior change, Diseases, Health care, Medical research, Psychology, Psychology, Risk factors

## Abstract

To identify psychosocial factors associated with medication adherence among elderly patients with chronic diseases, guided by the Integrated Theory of Health Behavior Change (ITHBC). A cross-sectional survey was conducted among 1,138 community-dwelling adults aged sixty years or older who were receiving long-term treatment for chronic diseases. A structured questionnaire was administered that incorporated validated instruments including the Adherence to Refills and Medications Scale (ARMS), the Medication Literacy Knowledge–Attitudes–Practices (KAP) Scale, the Beliefs about Medicines Questionnaire (BMQ), the Self-efficacy for Appropriate Medication Use Scale (SEAMS), and the Social Support Rating Scale (SSRS). Descriptive statistics, group comparisons, and binary logistic regression were used to examine the psychosocial factors associated with medication adherence. A total of 1,102 valid responses were obtained, yielding a response rate of 96.84%. Among the participants, 46.64% demonstrated good medication adherence. Guided by the ITHBC framework, psychosocial variables were categorized into intention-, capability-, and context-related domains. Logistic regression analysis showed that higher self-efficacy (OR = 0.874, *p* < 0.001) and higher medication literacy (OR = 0.980, *p* = 0.020) were associated with lower odds of poor medication adherence, whereas stronger medication concern beliefs (OR = 1.128, *p* = 0.008) and a history of adverse drug reactions (OR = 0.596, *p* = 0.035) were associated with poorer adherence. Compared with older adults living with a spouse, those living alone (OR = 3.848, *p* = 0.003) and those living with children (OR = 3.404, *p* = 0.004) showed significantly higher odds of poor medication adherence. Medication adherence among older adults with chronic diseases was associated with medication literacy, self-efficacy, social support, and medication beliefs. The ITHBC provided a useful framework for understanding these psychosocial determinants. The findings highlight the importance of multidimensional adherence-support interventions integrating medication education, psychosocial support, and self-management enhancement for older adults with chronic diseases.

**Supplementary Information:**

The online version contains supplementary material available at https://doi.org/10.1038/s41598-026-58469-z.

## Introduction

Medication adherence is a cornerstone of chronic disease management and an important determinant of clinical outcomes, quality of life, and healthcare utilization among older adults^1–4^. Despite the well-established benefits of adherence, non-adherence remains a persistent global challenge, with estimates suggesting that approximately 50% patients with chronic diseases do not take their medications as prescribed^1^. Older adults are particularly vulnerable because multimorbidity, polypharmacy, and age-related functional decline commonly complicate treatment^2^. In China, the prevalence of chronic diseases among adults aged sixty years and older now exceeds 80%^5^. Previous large-scale cohort research in Chinese adults has shown that multimorbidity is associated with substantially increased mortality risk, with mortality risk rising as the number of chronic conditions increases^6^. In this context, improving medication adherence has become an urgent public health priority.

Growing evidence indicates that medication adherence is shaped by a complex array of psychosocial associated factors. These include cognitive and perceptual factors such as medication beliefs^7^, perceived necessity and concerns^7^, self-efficacy^8^, medication literacy^9^, social support^10^, and patient–provider communication^11^. These psychosocial variables may shape how patients understand their illness, evaluate treatment benefits and risks, build confidence in managing their regimens, and mobilize support to overcome daily barriers. A recent review concluded that psychosocial associated factors often outweigh biomedical factors in predicting adherence, particularly in older adults with multimorbidity, highlighting the need for theoretically informed approaches to understand these associated factors^12^.

A variety of conceptual frameworks have been used to explain adherence behavior. The Necessity–Concerns Framework proposes that medication adherence is driven by a personal evaluation of the perceived need for treatment relative to concerns about adverse effects^7^. Social Cognitive Theory emphasizes self-efficacy and behavioral regulation as core associated factors of health behavior^13^. The Health Belief Model^14^ and Theory of Planned Behavior^15^ have also been applied to medication adherence, focusing on perceived susceptibility, behavioral intention, and motivational processes. Although these frameworks provide valuable insights, many focus on a limited set of psychological constructs and do not fully integrate environmental and capability-related associated factors that are particularly relevant in older populations.

The Integrated Theory of Health Behavior Change (ITHBC) was developed to integrate motivational, cognitive, behavioral-regulatory, and contextual association with on health behavior change^16^. In the original ITHBC framework, “knowledge and beliefs” includes individuals’ understanding of health information as well as cognitive appraisals such as self-efficacy and outcome beliefs, while “self-regulation skills and abilities” emphasizes the behavioral capacity to enact and sustain health behaviors. Because medication adherence among older adults involves both cognitive appraisal and practical medication-management capability, some psychosocial constructs examined in this study may conceptually interact across domains. Therefore, the present study applied ITHBC as an organizing heuristic rather than as a strict confirmatory theoretical model. The theory proposes that health behavior change is shaped by three interrelated domains: knowledge and beliefs, self-regulation skills and abilities, and social facilitation. Because these domains align closely with the known psychosocial factors associated with medication adherence, ITHBC offers a potentially comprehensive lens through which to examine adherence behavior in elderly patients with chronic diseases.

Although psychosocial determinants of medication adherence have been increasingly recognized, most previous studies have examined individual factors independently or focused on specific chronic diseases rather than older adults with multimorbidity living in community settings^9,12,17,18^. Furthermore, relatively few studies have simultaneously evaluated multiple psychosocial determinants within an integrated behavioral framework, particularly in Chinese older populations. As a result, the relative contributions of intention-related, capability-related, and contextual psychosocial factors to medication adherence remain insufficiently understood.

To address these gaps, this study applied ITHBC as an organizing framework to examine psychosocial determinants of medication adherence among community-dwelling older adults with chronic diseases in China. Specifically, the study aimed to identify key psychosocial factors associated with medication adherence across intention-, capability-, and context-related domains, thereby providing evidence to support the development of theory-informed adherence interventions for older adults.

## Materials and methods

### Study design and ethics

A cross-sectional survey was conducted from March to August 2024 in ten urban communities in Beijing, Guangzhou, and Taizhou. Trained community pharmacists administered face-to-face structured interviews. Convenience sampling was used because pharmacists routinely interacted with older adults receiving long-term medication therapy, which supported feasible recruitment in the community.

The study consisted of three phases: (1) a systematic review to identify psychosocial factors influencing medication adherence based on the ITHBC framework; (2) development of a questionnaire incorporating these factors; (3) community-based data collection and analysis. The study protocol was reviewed and approved by the Ethics Committee of Beijing Tiantan Hospital, Capital Medical University (Approval No.: KY2024-408-02).

### Rationale for selection of chronic diseases

Hypertension, type 2 diabetes, coronary heart disease and stroke were selected because preliminary investigations showed they were the most common chronic conditions among community-dwelling older adults on long-term home-based pharmacotherapy. These conditions have established monitoring indicators and standardized regimens, and community pharmacists frequently manage these patients, facilitating recruitment and assessment.

### Sample size calculation

The required sample size was calculated using the formula for cross-sectional studies (n = Z²P(1 − P)/d²). Assuming a conservative prevalence of good adherence of 50%, a 95% confidence level (Z = 1.96), and a margin of error of 3%, the minimum sample size required was 1,067. To account for non-response, the target sample size was increased by 10%. Ultimately, 1,138 individuals were invited and 1,102 completed the survey, yielding a recruitment rate of 96.84%.

### Participant identification and recruitment

Community pharmacists identified eligible participants through health records and routine chronic disease follow-up lists. Eligible individuals were approached during clinic visits or home medication counselling. Study information was provided verbally and in writing, and written informed consent was obtained prior to participation.

### Eligibility criteria

Inclusion criteria were: age ≥ 60 years; diagnosis of at least one of the four target chronic diseases; receipt of home-based long-term medication therapy for at least six months; residence in the community for at least six months; ability to communicate verbally; and willingness to participate.

Exclusion criteria included cognitive impairment, psychiatric disorders, malignant tumours, severe communication difficulties or inability to complete the interview.

Eligibility was confirmed through medical record review and participant screening.

### Data collection

Data were collected through face-to-face structured interviews conducted by trained pharmacists. Before the formal survey, all interviewers received standardized training regarding study objectives, participant communication, questionnaire administration procedures, ethical considerations, and data recording requirements to ensure consistency across interviewers.

During data collection, all questionnaire items were administered verbatim according to a standardized interview protocol. Interviewers were instructed not to paraphrase questions or provide interpretive guidance unless participants requested clarification of wording. In such cases, only neutral explanations were provided without suggesting responses or influencing participant answers.

Completed questionnaires were reviewed on-site for completeness and consistency immediately after the interview. Data were independently entered into electronic Questionnaire Star application using a double-entry procedure and subsequently exported to SPSS version 26.0 for statistical analysis. Data validation and consistency checks were performed prior to analysis to minimize entry errors.

### Instruments and psychometric properties

The questionnaire comprised validated Chinese versions of the following instruments:

Medication Adherence was assessed using the Adherence to Refills and Medications Scale (ARMS). The total score ranges from 12 to 48, with lower scores indicating better adherence. A total score of ≥ 16 was defined as poor adherence, while a score < 16 was classified as good adherence. The Chinese version was validated with strong reliability (Cronbach’s α = 0.890)^19^.

Medication Literacy was evaluated using Medication Literacy Knowledge–Attitudes–Practices Scale (KAP), which was validated in Chinese older adults and demonstrated strong internal consistency (Cronbach’s α = 0.918)^20^.

Medication Beliefs were measured using the Beliefs about Medicines Questionnaire (BMQ), which was originally developed by Horne et al.^21^ to assess patients’ cognitive representations of medications. The Chinese version of the BMQ has since been widely applied in Chinese populations and demonstrated acceptable applicability and psychometric utility in studies of medication adherence^22^.

Self-Efficacy was assessed with the Self-Efficacy for Appropriate Medication Use Scale (SEAMS). This 13-item scale uses a 1 to 3 scoring system per item, resulting in a total score of up to 39. Higher scores reflect greater self-efficacy for appropriate medication use. The Chinese version was validated with Cronbach’s α = 0.768^23^.

Social Support was evaluated using the Social Support Rating Scale (SSRS). This 10-item scale covers three dimensions: objective support (3 items), subjective support (4 items), and utilization of social support (3 items)^24^. The total score ranges from 0 to 40, with higher scores indicating a higher level of perceived social support. A total score < 22 was defined as insufficient social support, and a score ≥ 22 was classified as good social support.

All instruments used in this study were previously validated Chinese versions with established psychometric properties reported in earlier studies. No modifications were made to the original instrument items in the present study. To further assess internal consistency reliability within the current sample, Cronbach’s α coefficients were calculated for each scale, with all values exceeding 0.80, indicating good reliability.

Prior to the formal survey, a pilot test was conducted among 16 older adults with chronic diseases to evaluate the clarity, comprehensibility, and feasibility of the questionnaire administration procedures. Minor wording adjustments were made to improve readability and interview flow; however, no substantive modifications were made to the instrument content. Data obtained during the pilot phase were not included in the final statistical analysis.

### Alignment with the ITHBC framework

Questionnaire selection and variable classification were informed by the three domains proposed in the ITHBC: knowledge and beliefs, self-regulation skills and abilities, and social facilitation^16^. However, because the original ITHBC framework conceptualizes self-efficacy as part of the “knowledge and beliefs” domain, clarification of construct operationalization was necessary in the present study.

In this study, self-efficacy was operationally grouped within the “self-regulation skills and abilities” domain because the SEAMS instrument primarily assesses patients’ confidence in performing concrete medication-management behaviors under challenging circumstances, such as remembering doses, managing complex regimens, or taking medicines despite disruptions. Therefore, the construct was treated as reflecting behavioral capability and self-management capacity rather than solely cognitive belief.

Medication literacy, medication beliefs, and self-efficacy were recognized as conceptually related but distinct constructs. Medication literacy reflects the ability to obtain, understand, evaluate, and apply medication-related information; medication beliefs reflect cognitive appraisal of medication necessity and concerns; and self-efficacy reflects confidence in performing medication-taking behaviors. Although these constructs may associate with one another, they represent different psychosocial dimensions relevant to adherence behavior.

Accordingly, the ITHBC was used in this study as a flexible conceptual framework to organize psychosocial factors associated with medication adherence, rather than as a rigid structure requiring one-to-one correspondence between theoretical constructs and measurement instruments.

### Statistical analysis

Data were analyzed using SPSS version 26.0. Descriptive statistics summarized demographic and clinical characteristics. Independent-samples *t* tests and chi-square tests were used for univariate analysis. Variables included in the multivariable logistic regression model were selected based on ITHBC, prior literature, and clinical relevance. All theoretically relevant psychosocial variables were entered simultaneously into the multivariable model using the enter method. To assess potential multicollinearity among psychosocial constructs, variance inflation factors (VIFs) and correlation coefficients were examined before model fitting. All VIF values were below 5, indicating no serious multicollinearity (Supplementary Table S1). Pearson correlation coefficients among psychosocial predictors ranged from 0.022 to 0.440, all below the commonly accepted threshold of 0.70 (Supplementary Table S2). Odds ratios (ORs) and 95% confidence intervals (CIs) were reported.

In the regression analysis, the dependent variable was medication adherence status derived from the ARMS scale. “Poor adherence” (ARMS ≥ 16) was coded as 1, whereas “good adherence” (ARMS < 16) was coded as 0. Therefore, ORs > 1 indicate an increased likelihood of poor adherence, whereas ORs < 1 indicate a reduced likelihood of poor adherence.

## Results

### Integration and classification of psychosocial factors

**Fig. 1:**
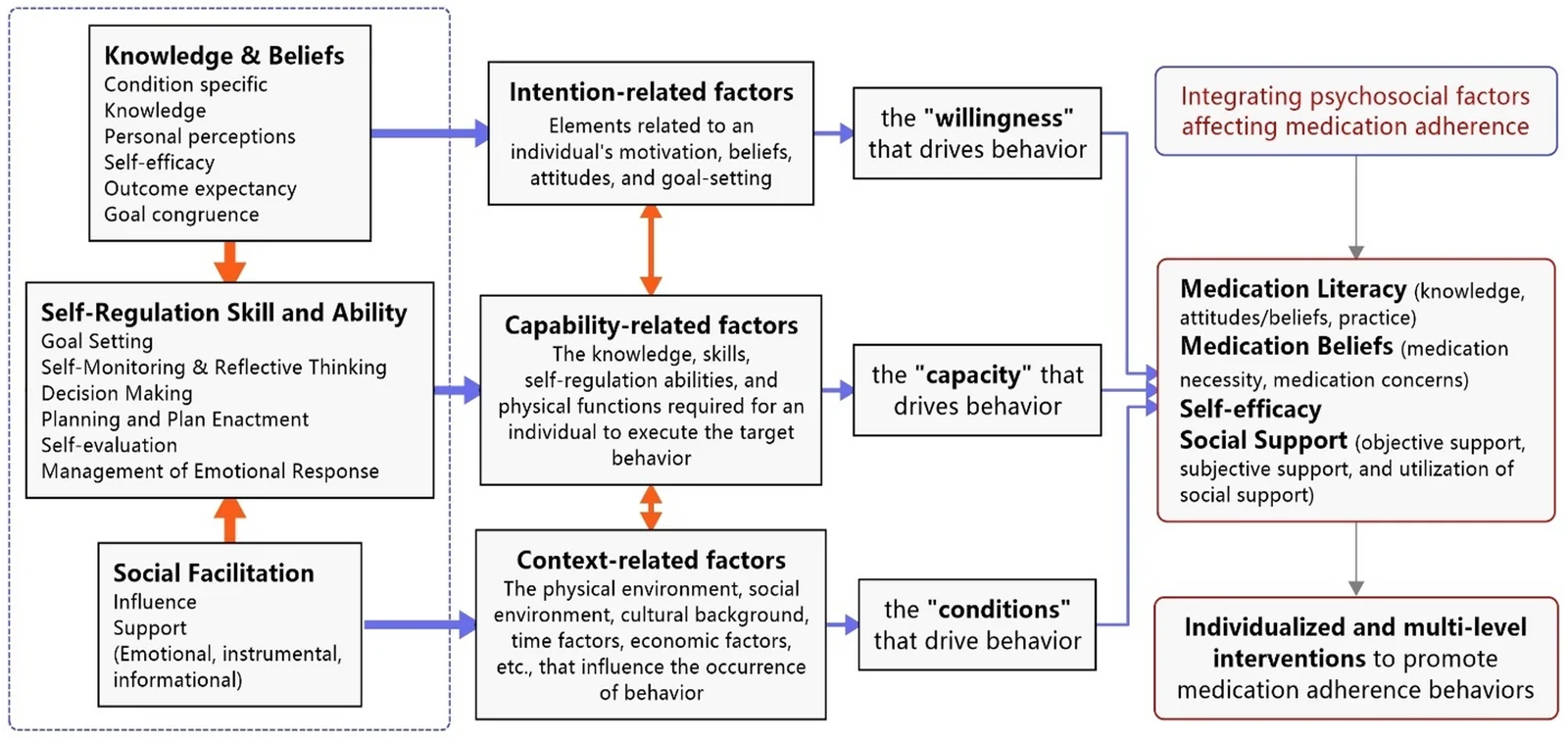
Integrating psychosocial factors associated with medication adherence based on ITHBC.

Guided by the ITHBC framework, psychosocial factors associated with medication adherence were categorized into intention-, capability- and context-related domains. Figure 1 illustrates how medication literacy, medication beliefs, self-efficacy and social support were mapped to these domains and integrated into the conceptual model. This classification provided the basis for subsequent quantitative analysis.

Table 1 presents the detailed categorization of psychosocial factors. All variables were grouped into three domains: (1) intention-related factors, including medication literacy, necessity beliefs and concern beliefs; (2) capability-related factors, including self-efficacy; and (3) context-related factors, including social support, family support and living arrangement. This classification served as the conceptual basis for the subsequent analysis.

**Table 1:** Measured psychosocial factors associated with medication adherence among older adults with chronic diseases organized by ITHBC domains.

ITHBC domain	Psychosocial factor	Tailored description for older adults with chronic diseases
Knowledge & Beliefs (Intention domain)	Medication literacy	Ability to understand dosing schedules, recognize side associations, judge when medication is working/not working, and interpret instructions in the context of multimorbidity and polypharmacy.
	Necessity beliefs	Older adults’ perception of the importance of long-term medication for controlling chronic conditions such as hypertension or diabetes, especially when symptoms are not obvious.
	Concern beliefs	Worries related to long-term toxicity, drug interactions, polypharmacy burden, and fear of adverse reactions due to age-related physiological changes.
Self-Regulation Skills & Abilities (Capability domain)	Self-efficacy	Confidence in managing complex medication regimens, remembering doses despite cognitive decline, and coping with difficulties such as multiple dosing times or adverse reactions.
Social Facilitation (Context domain)	Family support	Assistance from spouses or children with medication reminders, pharmacy pick-up, recognizing adverse reactions and providing emotional support.
	Social support	Support from neighbors, friends or community pharmacists, including counselling, education and follow-up.
	Living arrangement	Whether the patient lives alone or with family members who can assist with daily medication management.

Note: In the original ITHBC, self-efficacy is classified under knowledge and beliefs. However, in line with recent adherence literature and the operational needs of this study, we moved self-efficacy to the capability domain to better reflect its function as a self-regulatory enabler of consistent medication-taking behavior.

### Univariate analysis of medication adherence

Based on ARMS scoring criteria, 46.64% participants demonstrated good adherence and 53.36% demonstrated poor adherence. Results from chi-square tests and independent-samples *t* tests are presented in Tables 2 and 3.

As shown in Table 3, several psychosocial variables were significantly associated with medication adherence. Older adults with higher medication literacy and higher self-efficacy scores were more likely to demonstrate good medication adherence. Higher necessity belief scores and lower concern belief scores were also associated with better adherence. In addition, social support and living arrangement showed significant associations with medication adherence. Older adults living alone and those with lower levels of family involvement were more likely to exhibit poor medication adherence. Detailed multivariate regression results are presented in Table 4.

**Table 2:** Participants’ characteristics and univariate associations with medication adherence.

Variable	Characteristics	*n*	%	Medication adherence		χ^2^	*P*
				Good (*n* = 514)	Poor (*n* = 588)		
Gender	Male	541	49.09	253	288	0.006	0.936
	Female	561	50.91	261	300		
Age	60ཞ69	639	57.99	299	340	0.417	0.812
	70ཞ79	371	33.67	175	196		
	≥ 80	92	8.35	40	52		
Marital status	Married	914	82.94	426	488	0.626	0.890
	Single	19	1.72	9	10		
	Divorced	20	1.81	11	9		
	Widowed	149	13.52	68	81		
Educational level	Illiterate	80	7.26	30	50	28.539	***
	Primary school	271	24.59	100	171		
	Junior high school	313	28.40	143	170		
	Senior high school	233	21.14	127	106		
	Junior college	137	12.43	82	55		
	Undergraduate or above	68	6.17	32	36		
Smoking or not	Smoking	232	21.05	105	127	1.109	0.574
	No smoking	778	70.60	370	408		
	Quit smoking	92	8.35	39	53		
Drinking or not	Drinking	262	23.77	124	138	0.065	0.968
	No drinking	782	70.96	363	419		
	Quit drinking	58	5.26	27	31		
Living arrangement	Living alone	131	11.89	51	80	17.291	**0.001**
	With spouse	684	62.07	300	384		
	With children	245	22.23	138	107		
	Have a nanny/caregiver	42	3.81	25	17		
Employment status	Retired at home	829	75.23	383	446	0.343	0.843
	Re-employment or other work	94	8.53	44	50		
	Taking care of grandchildren	179	16.24	87	92		
Monthly income (CNY)	<1000	180	16.33	78	102	19.596	**0.001**
	1000 ~ 3000	259	23.50	102	157		
	3000 ~ 5000	371	33.67	168	203		
	5000 ~ 10,000	261	23.68	146	115		
	≥ 10,000	31	2.81	20	11		
Reimbursement type	Self-funded	26	2.36	19	7	11.346	**0.023**
	Medical insurance for works	574	52.09	275	299		
	Publicly-funded medical care	43	3.90	23	20		
	Medical insurance for urban and rural residents	459	41.65	197	262		
Course of chronic disease	<1 year	53	4.81	27	26	6.498	0.090
	1 ~ 5 year	240	21.78	97	143		
	5 ~ 10 year	281	25.50	127	154		
	≥ 10 year	528	47.91	263	265		
History of ADR	Never	865	78.49	426	439	13.792	**0.001**
	Yes, the patient can clearly describe	120	10.89	51	69		
	Yes, but the description was not clear	117	10.62	37	80		
Medication Beliefs	Positive Medication Beliefs	807	73.23	417	390	30.652	***
	Negative Medication Beliefs	295	26.77	97	198		
Necessity Beliefs	High Necessity	973	88.29	460	513	1.342	0.247
	Low Necessity	129	11.71	54	75		
Concern Beliefs	High Concerns	546	49.55	205	341	35.982	***
	Low Concerns	556	50.45	309	247		
Social Support Level	High Level	309	28.04	190	119	38.058	***
	Medium Level	770	69.87	315	455		
	Low Level	23	2.09	9	14		

Note: ADR, adverse drug reactions; *** represents *P*<0.001.

**Table 3:** Associations between psychosocial factors and medication adherence (continuous variables).

Variable	Total ($\bar{x} \pm S$	Medication adherence ($\bar{x} \pm S$± S)		t	*P*
		Good (*n* = 514)	Poor (*n* = 588)		
Age	69.99 ± 7.04	69.99 ± 6.89	69.99 ± 7.18	0.010	0.992
BMI (kg/m^2^)	24.68 ± 3.52	24.35 ± 3.46	24.96 ± 3.55	2.850	**0.004**
Number of chronic diseases	2.21 ± 1.16	2.16 ± 1.17	2.25 ± 1.14	-1.353	0.176
Number of medications	3.82 ± 2.75	3.67 ± 2.73	3.95 ± 2.77	-1.710	0.088
Medication beliefs	4.97 ± 5.85	6.56 ± 5.93	3.57 ± 5.41	8.699	***
Medication necessity	19.27 ± 4.35	20.12 ± 4.62	18.53 ± 3.96	6.164	***
Medication concerns	14.30 ± 4.48	13.56 ± 5.01	14.96 ± 3.86	-5.228	***
Self-efficacy	27.88 ± 7.42	31.55 ± 6.81	24.66 ± 6.37	17.259	***
Social support	39.65 ± 7.81	41.22 ± 7.96	38.29 ± 7.42	6.332	***
Objective support	8.69 ± 2.47	8.75 ± 2.49	8.63 ± 2.45	0.742	0.458
Subjective support	23.45 ± 5.15	24.47 ± 5.17	22.55 ± 4.98	6.253	***
Availability of support	7.52 ± 2.33	8.01 ± 2.55	7.10 ± 2.02	6.506	***
Medication literacy	195.74 ± 32.00	205.54 ± 33.64	187.18 ± 27.83	9.793	***
Medication knowledge	92.36 ± 19.58	97.36 ± 20.61	88.00 ± 17.53	8.054	***
Medication attitude	33.83 ± 6.58	35.52 ± 7.12	32.35 ± 5.67	8.092	***
Medication practice	69.55 ± 10.73	72.67 ± 10.58	66.82 ± 10.11	9.372	***

Note: BMI, Body Mass Index; *** represents *P*<0.001.

### Multivariate analysis of factors associated with medication adherence

Prior to regression analysis, multicollinearity diagnostics were performed for psychosocial variables including medication literacy, medication beliefs, concern beliefs, self-efficacy, and social support. Correlation coefficients between independent variables were all below 0.70, and VIF values ranged from 1.11 to 1.42 (Supplementary Table S1 and Table S2), suggesting acceptable independence among predictors and no evidence of problematic multicollinearity.

Variables selected based on the ITHBC framework, prior literature, and clinical relevance were included in the multivariable logistic regression model (Table 4). In the multivariable logistic regression analysis, poor medication adherence was treated as the outcome event (coded as 1).

Higher self-efficacy (OR = 0.874, 95% CI: 0.853–0.896, *p* < 0.001) and greater medication literacy (OR = 0.980, 95% CI: 0.964–0.997, *p* = 0.020) were associated with lower odds of poor adherence. Similarly, higher social support was associated with reduced odds of poor adherence (OR = 0.912, 95% CI: 0.847–0.983, *p* = 0.016).

In contrast, stronger medication concern beliefs were associated with increased odds of poor medication adherence (OR = 1.128, 95% CI: 1.082–1.175, *p* < 0.001). Participants with a clearly recognized history of adverse drug reactions had lower odds of poor adherence compared with those without such recognition (OR = 0.596, 95% CI: 0.368–0.965, *p* = 0.035).

Compared with older adults living with a spouse, those living alone (OR = 3.848, 95% CI: 1.569–9.441, *p* = 0.003) and those living with children (OR = 3.404, 95% CI: 1.487–7.793, *p* = 0.004) showed significantly higher odds of poor medication adherence.

**Table 4:** Multivariable logistic regression analysis of factors affecting medication adherence.

Variable	B	SE	OR	95%CI	*P*
Educational level (ref = illiterate)					
Primary school	0.319	0.464	1.376	0.554–3.418	0.491
Junior high school	0.362	0.388	1.436	0.671–3.071	0.351
Senior high school	-0.095	0.367	0.909	0.443–1.866	0.796
Junior college	-0.277	0.365	0.758	0.371–1.549	0.447
Undergraduate or above	-0.432	0.378	0.649	0.31–1.361	0.253
Living arrangement (ref = With spouse)					
Living alone	1.348	0.458	3.848	1.569–9.441	**0.003**
With children	1.225	0.423	3.404	1.487–7.793	**0.004**
Have a nanny/caregiver	0.556	0.431	1.744	0.750–4.057	0.196
BMI	0.046	0.022	1.048	1.004–1.093	**0.031**
Monthly income (ref = <1000¥)					
1000 ~ 3000¥	0.453	0.565	1.572	0.519–4.761	0.423
3000 ~ 5000¥	0.654	0.539	1.923	0.669–5.532	0.225
5000 ~ 10,000¥	0.634	0.507	1.884	0.697–5.095	0.212
≥ 10,000¥	0.4	0.496	1.491	0.565–3.939	0.420
Reimbursement type (ref = Self-funded)					
Medical insurance for works	-2.383	1.399	0.092	0.006–1.431	0.088
Publicly-funded medical care	-0.745	1.291	0.475	0.038–5.963	0.564
Medical insurance for urban and rural residents	-0.841	1.334	0.431	0.032–5.894	0.529
History of ADR (ref =Never)					
Yes, the patient can clearly describe	-0.518	0.246	0.596	0.368–0.965	**0.035**
Yes, but the description was not clear	-0.103	0.316	0.903	0.486–1.676	0.745
Medication beliefs	-0.037	0.018	0.964	0.930–1.000	**0.047**
Concern beliefs	0.155	0.033	1.128	1.082–1.175	**0.008**
Self-efficacy	-0.135	0.013	0.874	0.853–0.896	***
Social support	-0.092	0.038	0.912	0.847–0.983	**0.016**
Medication literacy	-0.02	0.009	0.980	0.964–0.997	**0.020**

Note: BMI, Body Mass Index; ADR, adverse drug reactions; *** represents *P*<0.001.

## Discussion

### Key findings and interpretations

This study examined a broad range of psychosocial factors associated with medication adherence among community-dwelling older adults with chronic diseases, using the ITHBC as a guiding organizational framework. Rather than evaluating the robustness of the ITHBC itself, the study used the theory to categorize psychosocial constructs into intention-, capability-, and context-related domains and to support the interpretation of how these domains relate to adherence behavior. The results therefore illustrate how selected ITHBC-aligned constructs function in this population, without implying validation of the full theoretical model.

Self-efficacy emerged as one of the factors most strongly associated with medication adherence in the present study. This finding is consistent with previous studies among older adults with hypertension, diabetes, and multimorbidity, which have consistently shown that higher medication self-efficacy is associated with better adherence behaviors and chronic disease self-management^9,23,25,26^. The magnitude of the association observed in our study also aligns with prior evidence suggesting that self-efficacy plays a central role in patients’ confidence to manage complex medication regimens under challenging circumstances. The comparatively stronger association observed for self-efficacy suggests that confidence in medication management may play a more proximal role in adherence behavior than knowledge-based factors alone.

Previous intervention studies have demonstrated that self-efficacy can be improved through skills-based education, individualized medication counseling, motivational interviewing, and behavioral coaching programs^8,27–31^. These interventions may enhance patients’ confidence in remembering medications, overcoming barriers to adherence, and managing treatment-related concerns. Therefore, interventions targeting medication self-efficacy may represent an important strategy for improving adherence among older adults with chronic diseases.

The present study found that stronger medication concern beliefs were associated with higher odds of poor medication adherence, which is consistent with previous studies reporting that concerns regarding medication side effects, dependency, and long-term harm may negatively influence adherence behaviors^7,32^. Older adults with greater medication concerns may intentionally reduce or discontinue medications because concerns regarding adverse reactions and long-term dependency can outweigh perceived treatment necessity, particularly in the context of multimorbidity and polypharmacy. Such cognitive risk–benefit evaluations may be especially prominent among older adults who experience treatment fatigue or previous adverse drug events.

However, findings regarding medication-related beliefs and literacy remain inconsistent across studies. For example, previous research found that lower health literacy was not significantly associated with a greater number of medications among community-dwelling older adults^33,34^. Such discrepancies may reflect differences in study populations, healthcare systems, medication complexity, or measurement approaches. In older adults with chronic diseases, medication adherence may be influenced not only by literacy level itself but also by beliefs regarding medication necessity, concerns about adverse effects, and the availability of social and family support. Therefore, medication beliefs should be understood within a broader psychosocial and behavioral context rather than as isolated determinants.

The present study found that higher social support was associated with lower odds of poor medication adherence among older adults with chronic diseases. Similar findings have been reported among community-dwelling older adults in China and among patients with chronic diseases in other populations, where emotional and instrumental support were associated with improved medication-taking behaviors and greater self-management capacity^10,35^. Social support may facilitate adherence by improving medication reminders, emotional encouragement, and access to healthcare resources.

Medication literacy demonstrated a positive association with adherence, although its effect was smaller than that of self-efficacy. Importantly, “knowledge” in the context of medication literacy extends beyond understanding instructions. It includes awareness of treatment benefits, recognition of adverse reactions, comprehension of disease–treatment relationships, and knowledge of when a medication is or is not working^17^. These broader knowledge domains may be associated with adherence both directly and indirectly through their effects on beliefs, confidence and self-management^9,18^. The present findings highlight the need for educational interventions that address not only procedural knowledge but also evaluative understanding of medications.

The finding that older adults living alone or with children had higher odds of poor medication adherence compared with those living with a spouse may reflect differences in daily support, medication supervision, or functional independence. Spousal companionship may facilitate routine medication-taking behaviors and improve adherence among older adults^36^.

The findings of this study extend existing ITHBC-informed research by demonstrating how psychosocial factors associated with medication adherence manifest differently in older adults with chronic diseases living in stable community settings. Whereas previous work, such as studies conducted during the COVID-19 pandemic among Black adults with diabetes, highlighted stress, disrupted routines and reduced social support as primary barriers^37^, our results emphasize functional capacity, medication concerns and household living structure as the more salient factors in an aging population. This contrast underscores the need to contextualize ITHBC applications to specific demographic and cultural groups. In addition, unlike studies that have used ITHBC to design or evaluate interventions, the present research employed the framework as an organizing structure to systematically examine a comprehensive set of psychosocial variables. By analyzing these factors simultaneously within a large community-based sample, the study provides new evidence on their relative importance, showing self-efficacy as the strongest predictor, followed by medication concerns and medication literacy, and offers nuanced insights that can support the prioritization of intervention targets for older adults with chronic conditions.

### Implications for practice and research

Based on these findings, several directions for targeted adherence interventions can be considered. First, interventions aiming to strengthen self-efficacy, such as pharmacist-led coaching, simplified dosing plans or skill-building support for managing complex regimens, may be particularly impactful given that self-efficacy emerged as the strongest associated factor. Second, addressing medication concerns through structured counseling, risk–benefit discussions and improved monitoring of adverse reactions may help reduce intentional non-adherence driven by fear or uncertainty. Third, enhancing medication literacy through personalized education materials, visual aids and regular follow-up can support older adults who face cognitive or comprehension barriers. Finally, because living arrangement and social support were meaningful associated variables, community-based support networks or home-visit programs may be especially helpful for older adults who live alone.

The present study contributes to the growing literature on medication adherence in older adults by applying the ITHBC to examine multiple psychosocial determinants within a unified framework. Compared with previous studies focusing on isolated predictors, our findings provide a more comprehensive understanding of how medication literacy, self-efficacy, social support, and medication beliefs collectively influence adherence behavior among older adults with chronic diseases.

By identifying modifiable psychosocial factors associated with poor adherence, the study also offers practical implications for clinicians, community healthcare providers, and policymakers. Integrating psychosocial assessment and adherence-support strategies into chronic disease management programs may help improve medication-taking behaviors and long-term health outcomes in aging populations.

### Strengths and limitations

A key strength of this study is its large sample and use of validated psychosocial instruments organized within a theoretical framework. Another strength is the integration of evidence synthesis, theory-guided variable selection, and community-based psychosocial assessment, which enabled a comprehensive evaluation of medication adherence–related factors in older adults with chronic diseases.

However, as a cross-sectional study, causal inference is limited, and the full behavioral progression outlined in the ITHBC including self-reflection could not be assessed. Although multicollinearity diagnostics did not indicate problematic overlap among psychosocial variables, moderate conceptual associations between constructs such as medication literacy, beliefs, and self-efficacy should still be considered when interpreting the regression estimates. The cross-sectional design precludes causal inference and limits conclusions regarding temporal directionality between psychosocial factors and medication adherence. Several observed associations may be bidirectional in nature. Longitudinal or intervention studies are needed to clarify causal pathways and dynamic interactions among these variables over time. Because data were collected through interviewer-administered questionnaires, the possibility of interviewer influence or social desirability bias cannot be completely excluded, despite standardized interviewer training and administration procedures. These limitations should be considered when interpreting the findings. Future research should explore how ITHBC-aligned constructs interact over time and how interventions targeting multiple domains can support sustained adherence in older adults.

## Conclusion

This study identified several psychosocial determinants of medication adherence among older adults with chronic diseases within the framework of ITHBC. Higher medication literacy, stronger self-efficacy, greater social support, and stronger necessity beliefs were associated with better medication adherence, whereas stronger medication concern beliefs and adverse drug reaction experiences were associated with poorer adherence.

These findings highlight the importance of incorporating psychosocial assessment into routine chronic disease management for older adults. Healthcare professionals should pay particular attention to patients with low medication literacy, insufficient social support, and negative medication beliefs, as these individuals may be at higher risk for poor adherence.

The findings also provide practical implications for intervention development and health policy. Future adherence-promoting interventions should adopt multidimensional strategies that integrate medication education, self-efficacy enhancement, family and social support strengthening, and counseling to address medication concerns. Community-based and primary healthcare programs targeting psychosocial barriers may help improve long-term medication adherence and chronic disease outcomes among older adults.

## Supplementary Information

Below is the link to the electronic supplementary material.

## Data Availability

The datasets used and/or analysed during the current study available from the corresponding author on reasonable request.
