# Supplementary material for: Psychosocial factors associated with medication adherence among older adults with chronic diseases: an ITHBC-informed cross-sectional study
Source: Sci Rep. 2026 Jun 16;16:27601. doi: 10.1038/s41598-026-58469-z (PMC13538693; doi:10.1038/s41598-026-58469-z)
Supplement: Supplementary file 1 [file 41598_2026_58469_MOESM1_ESM.docx]

**Supplementary Table S2**. Correlation matrix among psychosocial variables

|  | Medication beliefs | Self-efficacy | Social support | Medication literacy |
| --- | --- | --- | --- | --- |
| Medication beliefs | 1.000 | .385** | 0.022 | .206** |
| Self-efficacy | .385** | 1.000 | .225** | .440** |
| Social support | 0.022 | .225** | 1.000 | .281** |
| Medication literacy | .206** | .440** | .281** | 1.000 |

note：n=1102，*p<0.05，**p<0.01.
