# Supplementary material for: Psychosocial factors associated with medication adherence among older adults with chronic diseases: an ITHBC-informed cross-sectional study
Source: Sci Rep. 2026 Jun 16;16:27601. doi: 10.1038/s41598-026-58469-z (PMC13538693; doi:10.1038/s41598-026-58469-z)
Supplement: Supplementary file 2 [file 41598_2026_58469_MOESM2_ESM.docx]

**Supplementary Table S1**. Multicollinearity diagnostics among psychosocial predictors

| **Variable** | **Tolerance** | **VIF** |
| --- | --- | --- |
| Medication beliefs | 0.845 | 1.184 |
| Self-efficacy | 0.702 | 1.424 |
| Social support | 0.902 | 1.109 |
| Medication literacy | 0.769 | 1.301 |

Note: Tolerance values >0.10 and VIF values <5 indicate no significant multicollinearity among predictors.
